# Survival impact of prophylactic cranial irradiation in small-cell lung cancer in the modern era of magnetic resonance imaging staging

**DOI:** 10.1186/s13014-022-01994-8

**Published:** 2022-02-05

**Authors:** Yu Jin Lim, Changhoon Song, Hak Jae Kim, Korean Association for Lung Cancer, Korea Central Cancer Registry

**Affiliations:** 1 Department of Radiation Oncology, Kyung Hee University College of Medicine, Kyung Hee University Medical Center, Seoul, Republic of Korea; 2 Department of Radiation Oncology, Seoul National University College of Medicine, Seoul National University Bundang Hospital, 82, Gumi-ro 173Beon-gil, Bundang-gu, Seongnam, 13620 Republic of Korea; 3 Korean Association for Lung Cancer, Seoul, Republic of Korea; 4 Department of Radiation Oncology, Seoul National University College of Medicine, 101 Daehak-ro, Jongno-gu 03080, Seoul, Republic of Korea; 5 Cancer Research Institute, Seoul National University College of Medicine, Seoul, Republic of Korea

**Keywords:** Small-cell lung cancer, Prophylactic cranial irradiation, Brain metastasis, Magnetic resonance imaging, Limited-stage, Extensive-stage

## Abstract

**Background:**

In the modern era of magnetic resonance imaging (MRI) staging, the benefit of prophylactic cranial irradiation (PCI) in patients with small-cell lung cancer (SCLC) has been controversial. This study evaluated the prognostic impact of PCI in patients with limited- or extensive-stage SCLC who had no brain metastases at diagnosis according to MRI.

**Methods:**

Data from newly diagnosed patients in 2014 from the Korean Association for Lung Cancer Registry database were used. Patients with limited- or extensive-stage SCLC who had no brain metastases according to MRI were identified. Univariate and multivariate survival analyses were conducted to assess the prognostic association of PCI.

**Results:**

Of 107 and 122 patients with limited- and extensive-stage SCLC, 24% and 14% received PCI, respectively. In the limited-stage SCLC group, the 2-year overall survival (OS) rates of patients who received PCI and those who did not were 50% and 29% (*P* = 0.018), respectively. However, there was no significant difference in OS for patients with extensive-stage SCLC (*P* = 0.336). After adjusting for other covariates, PCI was found to be associated with improved OS in the limited-stage SCLC group (*P* = 0.005). Based on the time-course hazard rate function plots in the limited-stage SCLC group, the OS benefit of PCI was maximized within the first year of follow-up.

**Conclusions:**

In the modern era of MRI staging, PCI might be beneficial for patients with limited-stage SCLC but not for those with extensive-stage SCLC. Further studies with a large sample size are needed to verify the prognostic association of PCI.

**Supplementary Information:**

The online version contains supplementary material available at 10.1186/s13014-022-01994-8.

## Introduction

According to recent data, lung cancer is the most fatal malignancy worldwide [1]. Small-cell lung cancer (SCLC) accounts for approximately 13.6% of all newly diagnosed lung cancer cases in Korea [2]. Considering that this type of cancer is aggressive, with rapid progression, approximately two-thirds of patients are diagnosed with extensive-stage SCLC (American Joint Committee on Cancer [AJCC] stage IV) [3]. Approximately 15‒33% of patients with SCLC have subclinical brain metastases upon initial diagnosis [4–6]. As the rate of early intracranial dissemination is relatively high, prophylactic cranial irradiation (PCI) has been an essential component in the management of SCLC [7].

The European Organisation for Research and Treatment of Cancer (EORTC) trial showed improvements in survival with intracranial prophylaxis, especially for patients with extensive-stage SCLC [8]. The use of PCI has been widely considered in extensive-stage SCLC. However, the trial has been criticized for not performing brain magnetic resonance imaging (MRI) after chemotherapy and prior to PCI [9]. The EORTC trial recommended the evaluation of the brain only for symptomatic patients. Later, in 2017, a Japanese study was conducted on patients who did not have brain metastases at diagnosis and after chemotherapy according to MRI [10]. According to the study, the use of PCI was not superior to regular follow-up in terms of overall survival (OS) when MRI was performed. As such, recent clinical guidelines have raised questions about whether PCI is required in current clinical settings where routine examination using MRI is accessible and commonly performed [11, 12]. In real-world clinical settings, a Dutch nationwide cohort study observed that the use of PCI has significantly declined in both limited- and extensive-stage SCLC since 2017 [13]. However, according to recent survey data among US radiation oncologists, approximately 98% of responders considered the application of PCI in both limited- and extensive-stage SCLC [14, 15]. Furthermore, according to the recent ASTRO Clinical Practice Guideline [3], shared decision-making on PCI versus MRI surveillance is strongly recommended with extensive-stage SCLC patients who respond to chemotherapy. In that guideline, PCI is strongly recommended only to patients with stage II-III limited-stage SCLC who are less than 70 years of age with good performance status and respond to initial therapy.

There is less controversy regarding the efficacy of PCI after first-line therapy in patients with limited-stage disease than in those with extensive-stage disease [16]. Several meta-analyses have shown that PCI is beneficial for the survival of patients with limited-stage disease who respond to initial therapy [17–19]. However, the aforementioned Japanese trial has raised questions regarding whether extensive-stage findings could be extrapolated to patients with limited-stage disease [20]. Further investigations should be conducted to determine whether the routine administration of PCI in patients with limited-stage disease is valid based on current clinical patterns.

This study evaluated the efficacy of PCI in patients with limited- or extensive-stage SCLC who had no brain metastases at diagnosis according to MRI. Based on multicenter cohort data from a Korean nationwide lung cancer registry database, the role of PCI in the contemporary era was assessed.

## Materials and methods

### Database

We used data from the Korean Association for Lung Cancer Registry (KALC-R) database, which were collected through a retrospective sampling survey performed by the Korea Central Cancer Registry and the Lung Cancer Registration Committee [2]. The final data included data from 13 regional cancer centers and 39 hospitals in Korea, and the sample size of each center was determined by the probability of selection method, which was based on whether a significant number of patients were enrolled annually. According to predefined criteria, 2621 patients were registered using the systematic sampling method in 2014. Of the entire lung cancer population, patients with SCLC were selected and analyzed. This study protocol was reviewed and approved by the institutional review board of the National Cancer Center in Korea (NCC2018-0193), which waived the requirement for informed consent due to the retrospective nature of the study.

### Study population

**Fig. 1:**
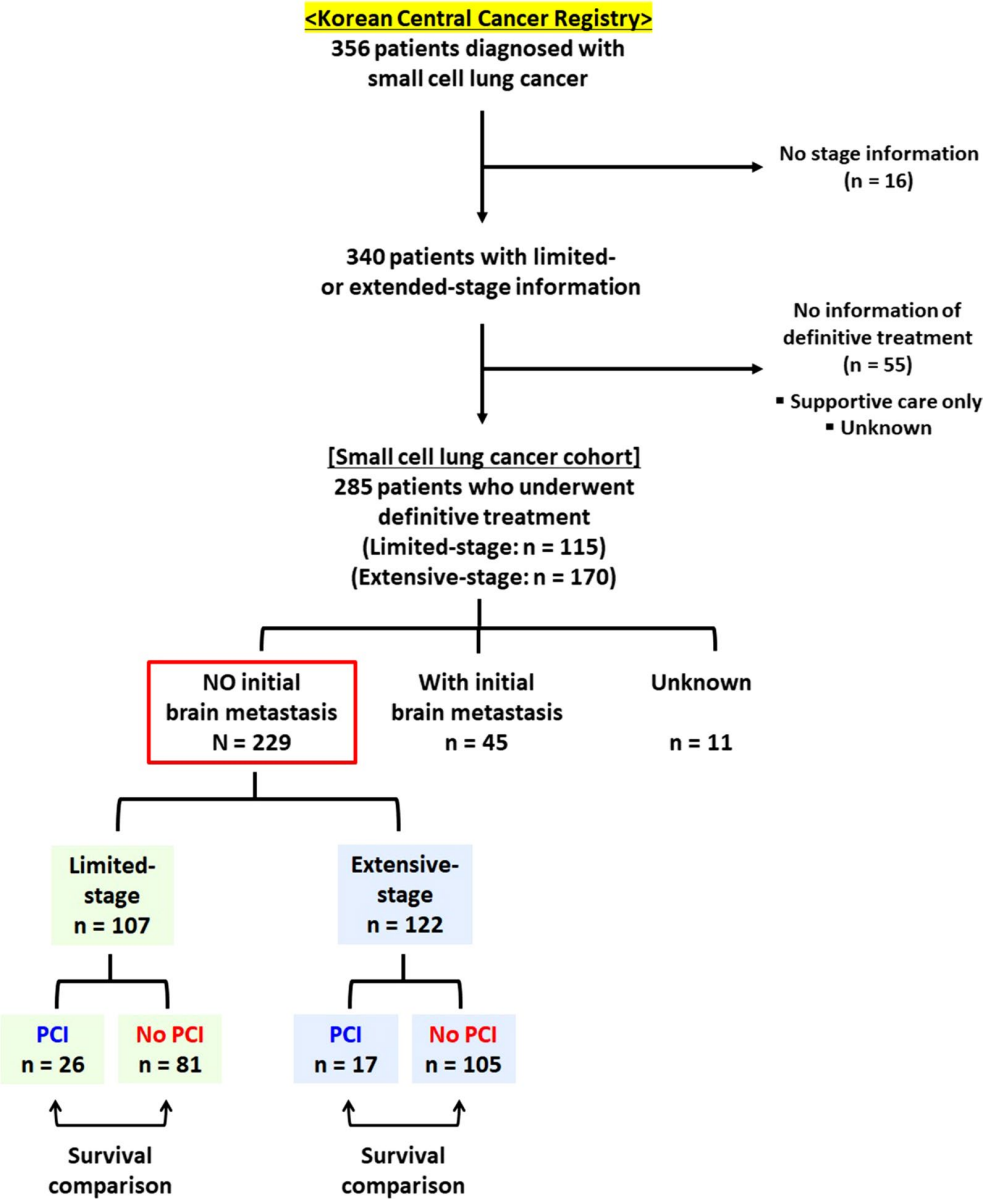
Flowchart of the patient selection process

Figure 1 represents the flowchart of the patient selection process. Of 356 patients with SCLC in the database, 71 were excluded due to a lack of information about limited or extensive staging and/or definitive treatment. In the SCLC cohort, 229 of 285 patients without brain metastases during the initial diagnosis received definitive therapy. The final patient cohort was stratified according to limited- and extensive-stage disease, and the survival outcomes of the two subsets of patients—with and without PCI treatment—in each stage group were compared.

Based on a standardized protocol, baseline variable data were collected for each patient. The patient-related information included age, sex, Eastern Cooperative Oncology Group (ECOG) performance score, body mass index (BMI) (kg/m^2^), the number of symptoms at initial diagnosis, and smoking history. The tumor-related characteristics were the histopathological type, the date of the pathological diagnosis, extrathoracic metastases, and the clinical TNM stage according to the seventh edition of the AJCC staging system. Information on the Veterans Administration Lung Study Group (VALSG) two-stage classification scheme was also collected for the SCLC patients. Each patient underwent brain MRI at baseline based on the nationwide assessment program for appropriate lung cancer diagnosis and treatment. Information regarding treatment, such as the first-line therapy and subsequent chemotherapy, radiotherapy (RT), and surgery, included the purpose, start and end date, regimens, number of cycles, radiation dose fractionation, sites of the radiation field, treatment modality, extent of surgical resection, and completeness of resection. Clinical follow-up was continued until December 2018.

### Statistical analysis

To compare baseline characteristics between the two groups, a paired t test or the Mann‒Whitney U test was used to assess continuous variables. Pearson’s chi-square test or Fisher’s exact test was used to evaluate categorical variables. The primary outcome of interest was overall survival (OS), which was defined as the time between the date of histological diagnosis and death. Using a log-rank test, Kaplan‒Meier analysis was performed to compare survival outcomes according to patient-, tumor-, and treatment-related factors. In the multivariate analysis, Cox proportional hazards models were used. Multicollinearity was not observed for each variable included in the multivariate analysis. Two-tailed *P* values of < 0.05 were considered statistically significant. All statistical analyses were conducted using SPSS 18 (IBM, Armonk, NY) and R version 4.0.2 (R Foundation for Statistical Computing, Vienna, Austria).

## Results

### Patient characteristics

There were 229 patients with SCLC who did not have brain metastases at diagnosis. In total, 107 and 122 patients were diagnosed with limited- and extensive-stage SCLC, respectively. Table 1 describes the clinicopathological and treatment-related characteristics according to disease classification. The median patient age (68 years old in both groups, *P* = 0.923), BMI (23.8 and 23.0 kg/m^2^, *P* = 0.640), and number of symptoms at diagnosis (one for both groups, *P* = 0.713) were comparable between the limited- and extensive-stage SCLC groups. In addition, there was no significant difference between groups in terms of the sex distribution (*P* = 0.966) or ECOG performance score (*P* = 0.393). The proportions of patients with cT3‒4 (38% vs. 56%, *P* = 0.013) and cN2‒3 (79% vs. 90%, *P* < 0.001) disease were lower in the limited-stage SCLC group than in the extensive-stage disease group. The extensive-stage SCLC group was more commonly treated with chemotherapy alone than the limited-stage SCLC group (81% vs. 35%), and chemoradiotherapy (CRT) was less likely in the extensive-stage SCLC group (12% vs. 53%) (*P* < 0.001). Treatment details about the first-line chemotherapy, thoracic RT, and salvage treatment at tumor relapse are shown in Additional file 1: Supplementary Materials.

**Table 1:** Baseline characteristics of small-cell lung cancer study population (N = 229)

Variables	Number of patients (%)		*P*
	Limited-stage (n = 107)	Extensive-stage (n = 122)	
*Age (year)*			
Median (range)	68 (43‒82)	68 (32‒91)	0.923
*Gender*			
Male	91 (85)	104 (85)	0.966
Female	16 (15)	18 (15)	
*ECOG performance score*			
0‒1	76 (71)	82 (67)	0.393
≥ 2	11 (10)	20 (16)	
Unknown	20 (19)	20 (16)	
*Ever-smoker*			
Yes	95 (89)	93 (76)	0.032
No	10 (9)	27 (22)	
Unknown	2 (2)	2 (2)	
*Body mass index (kg/m*^*2*^*)*			
Median (range)	23.8 (15.4‒32.7)	23.0 (17.1‒32.8)	0.640
*No. of symptoms at diagnosis*			
Median (range)	1 (0‒4)	1 (0‒5)	0.713
*Clinical T stage*			
T1	15 (14)	5 (4)	0.013
T2	29 (27)	22 (18)	
T3	11 (10)	21 (17)	
T4	30 (28)	48 (39)	
Unknown	22 (21)	26 (21)	
*Clinical N stage*			
N0	11 (10)	2 (2)	< 0.001
N1	9 (8)	3 (2)	
N2	42 (39)	35 (29)	
N3	43 (40)	74 (61)	
Unknown	2 (2)	8 (6)	
*Definitive treatment*			
Chemoradiotherapy^a^	57 (53)	15 (12)	< 0.001
Chemotherapy	37 (35)	99 (81)	
Radiotherapy	6 (6)	5 (4)	
Surgery (± adjuvant therapy)	7 (6)	3 (3)	
*Prophylactic cranial irradiation*			
Yes	26 (24)	17 (14)	0.045
No	81 (76)	105 (86)	
*Chemotherapy regimens*			
Cisplatin-based doublet	67 (63)	66 (54)	0.576
Carboplatin-based doublet	27 (25)	37 (30)	
Others	3 (3)	3 (3)	
Not available	10 (9)	16 (13)	
*Cycles of first-line chemotherapy*			
< 4	28 (26)	38 (31)	0.380
≥ 4	69 (65)	68 (56)	
Not available	10 (9)	16 (13)	
*Salvage treatment*			
Chemoradiotherapy^a^	15 (14)	18 (15)	0.910
Chemotherapy	16 (15)	22 (18)	
Others^b^	10 (9)	12 (10)	
No treatment	66 (62)	70 (57)	

^a^Cases with concurrent or sequential chemoradiotherapy were included

^b^Patients who received radiotherapy alone or surgery (± adjuvant therapy) were included

ECOG, Eastern Cooperative Oncology Group

### PCI

In total, 26 (24%) and 17 (14%) patients with limited- and extensive-stage SCLC were treated with PCI, respectively. The median daily and total radiation doses given to the limited- and extensive-stage SCLC groups were 2.5 (range 1.5‒3 and 2‒5) Gy and 25 (range 25‒37.5 and 20‒35) Gy, respectively. Most patients received PCI with the conventional, three-dimensional conformal, or intensity-modulated RT technique (24 and 15 in the limited- and extensive-stage SCLC groups, respectively).

### Distribution of variables according to PCI treatment

As shown in Table 2, the median age of patients with limited-stage SCLC who were not treated with PCI (non-PCI group) was higher than that of patients who were treated with PCI (PCI group) (69 vs. 60 years, *P* = 0.001). The differential predominance of CRT (73% vs. 47%) and chemotherapy alone (23% vs. 38%) was marginally significant between the PCI group and the non-PCI group (*P* = 0.055). The proportion of patients who received ≥ 4 cycles of first-line chemotherapy was higher in the PCI group than in the non-PCI group (96% vs. 62%, *P* = 0.001). However, there was no significant difference in terms of other clinical and treatment-related variables.

**Table 2:** Clinical and treatment-related characteristics of patients with limited-stage who had no brain metastases at baseline

Variables	Number of patients (%)		*P*
	PCI (n = 26)	No PCI (n = 81)	
*Age (year)*			
Median (range)	60 (43‒76)	69 (44‒82)	0.001
*Gender*			
Male	22 (85)	69 (85)	1.000
Female	4 (15)	12 (15)	
*ECOG performance score*^*a*^			
0‒1	21 (81)	55 (68)	1.000
≥ 2	3 (12)	8 (10)	
*Ever-smoker*^*a*^			
No	1 (4)	9 (11)	0.445
Yes	25 (96)	70 (86)	
*Body mass index (kg/m*^*2*^*)*			
Median (range)	24.1 (15.8‒29.4)	23.8 (15.4‒32.7)	0.776
*No. of symptoms at diagnosis*			
Median (range)	2 (0‒3)	1 (0‒4)	0.608
*Clinical T stage*^*a*^			
T1‒2	13 (56)	31 (50)	0.593
T3‒4	10 (44)	31 (50)	
*Clinical N stage*^*a*^			
N0‒1	2 (8)	18 (22)	0.147
N2‒3	23 (92)	62 (78)	
*Definitive treatment*			
Chemoradiotherapy^b^	19 (73)	38 (47)	0.055
Chemotherapy	6 (23)	31 (38)	
Others^c^	1 (4)	12 (15)	
*Chemotherapy regimens*			
Cisplatin-based doublet	19 (73)	48 (68)	0.551
Carboplatin-based doublet	6 (23)	20 (28)	
Others	1 (4)	3 (4)	
*Cycles of first-line chemotherapy*			
< 4	1 (4)	27 (38)	0.001
≥ 4	25 (96)	44 (62)	
*Salvage treatment*			
Chemoradiotherapy^b^	5 (19)	10 (12)	0.676
Chemotherapy	5 (19)	11 (14)	
Others^c^	2 (8)	8 (10)	
No treatment	14 (54)	52 (64)	

^a^Missing values were excluded

^b^Cases with concurrent or sequential chemoradiotherapy were included

^c^Patients who received radiotherapy alone or surgery (± adjuvant therapy) were included

PCI, prophylactic cranial irradiation; ECOG, Eastern Cooperative Oncology Group

In the extensive-stage SCLC group (Additional file 1: Table S1), there was a trend toward the differential use of PCI based on age, with marginal significance (*P* = 0.061). The proportion of patients treated with CRT was higher in the PCI group than in the non-PCI group (47% vs. 7%, *P* < 0.001). Patients who received PCI were more likely to receive aggressive salvage treatment with CRT or chemotherapy than those who did not receive PCI (*P* = 0.070).

### Prognostic associations between PCI and other variables

**Fig. 2:**
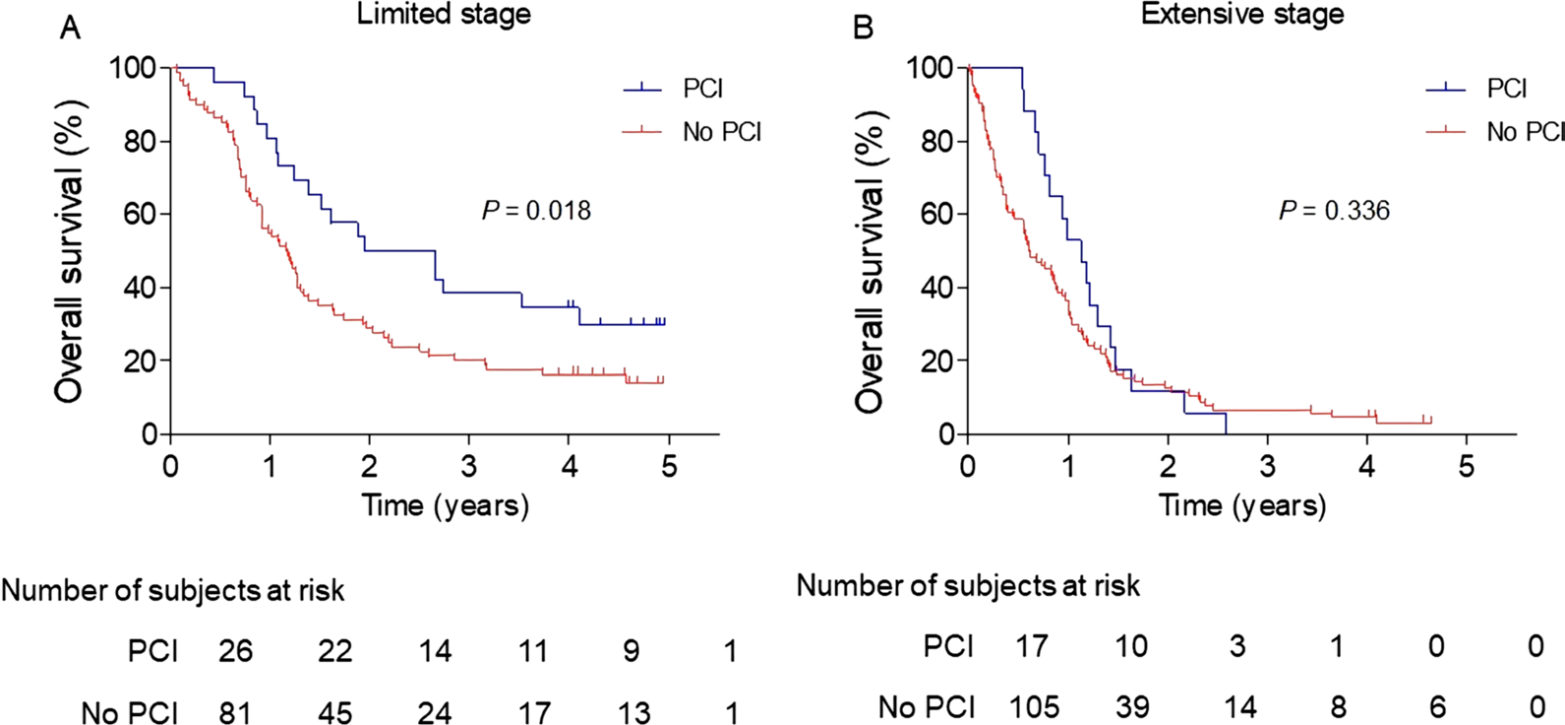
Overall survival in the **A** limited- and **B** extensive-stage small-cell lung cancer groups

Figure 2 shows the Kaplan‒Meier survival curves for OS. In the PCI and non-PCI groups, the median OS times were 23.4 (95% confidence interval [CI] 7.7‒39.1) and 13.9 (95% CI 10.8‒17.0) months for patients with limited-stage SCLC and 13.6 (95% CI 9.7‒17.5) and 7.3 (95% CI 4.4‒10.1) months for patients with extensive-stage SCLC, respectively. The 2-year OS rates for patients who received treatment with PCI and those who did not were 50% and 29% in the limited-stage SCLC group (*P* = 0.018) and 12% and 13% in the extensive-stage SCLC group (*P* = 0.336), respectively.

In a univariate analysis of patients with limited-stage SCLC (Table 3), age (≤ 68 vs. > 68 years, *P* = 0.043), BMI (≥ 23.6 vs. < 23.6 kg/m^2^, *P* = 0.020), the number of symptoms at diagnosis (0 vs. ≥ 1, *P* = 0.040), cT (T1‒2 vs. T3‒4, *P* = 0.038), cN (N0‒1 vs. N2‒3, *P* = 0.036), definitive treatment (CRT vs. chemotherapy alone and others, *P* < 0.001), and the administration of PCI (yes vs. no, *P* = 0.018) were associated with OS. Among these variables, ≥ 1 symptom at diagnosis (hazard ratio [HR] 2.48; 95% CI 1.19‒5.16), cN2‒3 (HR 3.01; 95% CI 1.25‒7.26), chemotherapy alone as a definitive treatment (HR 3.07; 95% CI 1.70‒5.56), and no administration of PCI (HR 2.46; 95% CI 1.30‒4.63) were significant poor prognostic factors in the multivariate analysis (Table 4).

**Table 3:** Univariate analysis for overall survival according to limited- and extensive-stage

Variables	Limited-stage			Extensive-stage		
	n (%)	2-Year rate (%)	*P*	n (%)	2-Year rate (%)	*P*
*Age (years)*^*a*^						
≤ 68	55 (51)	40	0.043	61 (50)	18	0.007
> 68	52 (49)	28		61 (50)	7	
*Gender*						
Male	91 (85)	31	0.095	104 (85)	12	0.766
Female	16 (15)	50		18 (15)	18	
*ECOG score*						
0‒1	76 (71)	41	0.223	82 (67)	15	< 0.001
≥ 2	11 (10)	27		20 (16)	5	
*Ever-smoker*						
Yes	95 (89)	32	0.604	93 (76)	7	0.068
No	10 (9)	56		27 (22)	14	
*Body mass index (kg/m*^*2*^*)*^*a*^						
≥ 23.6	55 (51)	44	0.020	50 (41)	18	0.021
< 23.6	43 (40)	21		64 (53)	10	
*No. of symptoms at diagnosis*						
0	27 (25)	48	0.040	26 (21)	23	0.255
≥ 1	80 (75)	29		96 (79)	10	
*Clinical T stage*						
T1‒2	44 (41)	41	0.038	27 (22)	15	0.777
T3‒4	41 (38)	29		69 (57)	12	
*Clinical N stage*						
N0‒1	20 (19)	50	0.036	5 (4)	60	0.066
N2‒3	85 (79)	30		109 (89)	11	
*Extrathoracic metastasis*						
No				32 (26)	19	0.147
Yes				90 (74)	10	
*Definitive treatment*						
Chemoradiotherapy^b^	57 (53)	44	< 0.001	15 (12)	27	0.072
Chemotherapy	37 (35)	11		99 (81)	10	
Others^c^	13 (12)	54		8 (7)	13	
*PCI*						
Yes	26 (24)	50	0.018	17 (14)	12	0.336
No	81 (76)	29		105 (86)	13	
*Salvage treatment*						
Chemoradiotherapy^b^	15 (14)	47	0.649	18 (15)	44	< 0.001
Chemotherapy	16 (15)	31		22 (18)	9	
Others^c^	10 (9)	40		12 (10)	17	
No treatment	65 (61)	31		70 (57)	4	

^a^The median value was used as the cutoff point

^b^Cases with concurrent or sequential chemoradiotherapy were included

^c^Patients who received radiotherapy alone or surgery (± adjuvant therapy) were included

ECOG, Eastern Cooperative Oncology Group; PCI, prophylactic cranial irradiation

**Table 4:** Multivariate analysis for overall survival in patients with limited-stage who had no brain metastases at baseline

Variables	Multivariate analysis		
	Hazard ratio	95% CI	*P*
*Age (years)*^*a*^			
≤ 68	Ref		
> 68	1.20	0.70‒2.05	0.520
*Body mass index (kg/m*^*2*^*)*^*a*^			
≥ 23.6	Ref		
< 23.6	1.41	0.82‒2.41	0.210
*No. of symptoms at diagnosis*			
0	Ref		
≥ 1	2.48	1.19‒5.16	0.015
*Clinical T stage*			
T1‒2	Ref		
T3‒4	0.78	0.43‒1.42	0.416
*Clinical N stage*			
N0‒1	Ref		
N2‒3	3.01	1.25‒7.26	0.014
*Definitive treatment*			
Chemoradiotherapy^b^	Ref		
Others^c^	1.14	0.41‒3.16	0.796
Chemotherapy	3.07	1.70‒5.56	< 0.001
*PCI*			
Yes	Ref		
No	2.46	1.30‒4.63	0.005

^a^The median value was used as the cutoff point

^b^Cases with concurrent or sequential chemoradiotherapy were included

^c^Patients who received radiotherapy alone or surgery (± adjuvant therapy) were included

CI, confidence interval; Ref, reference; PCI, prophylactic cranial irradiation

For patients with extensive-stage SCLC, different OS outcomes were observed according to age (≤ 68 vs. > 68 years, *P* = 0.007), ECOG performance score (0‒1 vs. ≥ 2, *P* < 0.001), BMI (≥ 23.6 vs. < 23.6 kg/m^2^, *P* = 0.021), cN (N0‒1 vs. N2‒3, *P* = 0.066), and salvage treatment (CRT vs. chemotherapy, others, and without treatment, *P* < 0.001) (Table 3). Of these factors, age > 68 years (HR 1.69; 95% CI 1.08‒2.64), an ECOG performance score of ≥ 2 (HR 2.11; 95% CI 1.21‒3.66), salvage chemotherapy alone (HR 2.66; 95% CI 1.27‒5.57), and not receiving any form of salvage treatment (HR 4.09; 95% CI 2.19‒7.62) were independent poor prognostic factors for OS (Additional file 1: Table S2).

### Time-course mortality risks of patients with limited-stage SCLC

**Fig. 3:**
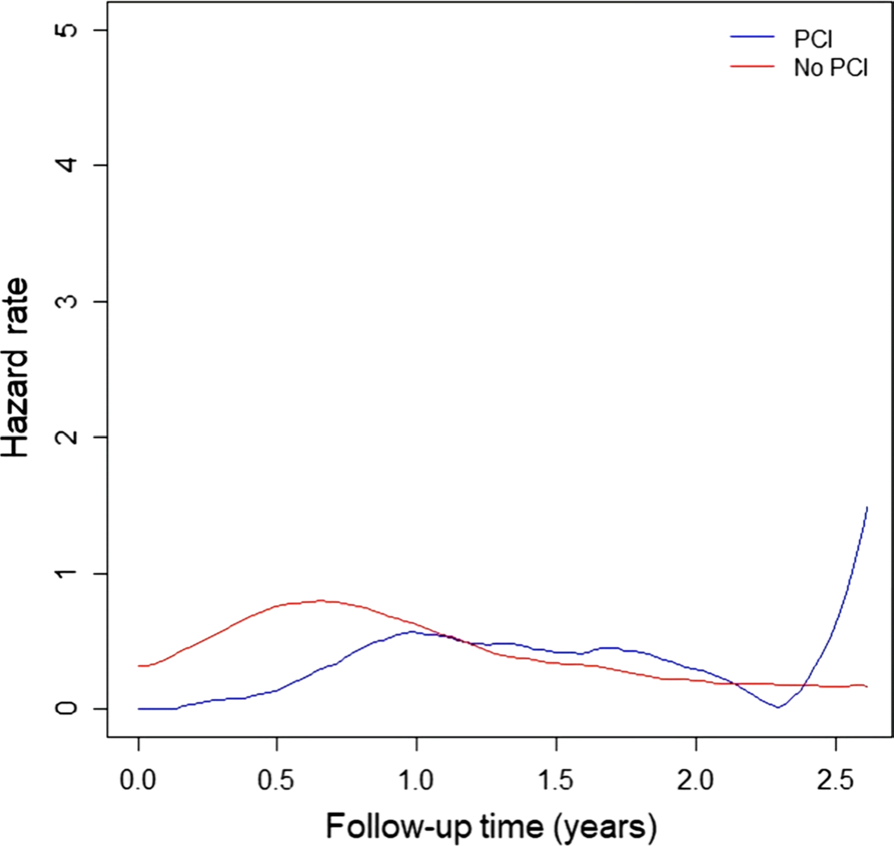
Time-course hazard rate function plots of overall mortality in patients with limited-stage small-cell lung cancer

Figure 3 shows the time-course hazard rate function plots of all-cause mortality in patients with limited-stage SCLC. During a 1-year follow-up, patients who were not treated with PCI had a greater risk of mortality than those who were treated.

## Discussion

In this nationwide cohort study, the prognostic impact of PCI in patients with SCLC who had no brain metastases at diagnosis differed according to disease classification (limited and extensive stage). For patients with limited-stage SCLC, PCI was associated with a more favorable OS in the univariate analysis and maintained a statistically significant difference in the multivariate analysis after adjusting for other clinical covariates. However, for patients with extensive-stage SCLC, there was no significant prognostic difference between the PCI and non-PCI groups. In the limited-stage SCLC group, the time-course hazard rate function plots for OS showed that patients who did not receive PCI had a higher mortality risk within the first year of follow-up. PCI might be beneficial for patients with limited-stage SCLC but not for those with extensive-stage SCLC.

In a recent observational study by Farris et al., PCI treatment was an independent predictor of better progression-free survival (PFS) in patients with limited-stage SCLC (median: 26.3 and 12.3 months in patients with and without PCI, respectively, *P* = 0.02) [20]. The study also included patients who had no brain metastases at diagnosis, and the benefit of PCI in patients with limited-stage disease was in accordance with our study results. Furthermore, several single-center retrospective studies have shown that PCI may not be beneficial for the survival of all patients with limited-stage disease [21–23]. One Japanese study did not show significant differences between the PCI and non-PCI groups in terms of OS (*P* = 0.54) or PFS (*P* = 0.72) [21]. An Italian retrospective study also reported that PCI did not seem to influence OS (*P* = 0.21) or PFS (*P* = 0.34) [22]. However, the current study used nationwide multicenter cohort data from the central lung cancer registry. The registry includes data from 52 institutions, including relatively accurate staging information, treatment details, and survival data. Thus, this population-based research can provide additional insights into the prognostic role of PCI in the contemporary era. Despite some limitations expected from the retrospective design, it was noteworthy that the favorable prognostic association of PCI treatment was suggested in patients with limited-stage SCLC, even after adjusting for other possible confounding effects.

Thus, a modern-day randomized trial of patients with limited- and extensive-stage diseases undergoing MRI staging and surveillance must be performed. In relation to this, the results of the recently embarked Southwest Oncology Group (SWOG) randomized phase III MAVERICK trial to evaluate MRI surveillance and PCI vs. MRI surveillance alone are eagerly awaited [24]. In Europe, the EORTC established a trial to assess PCI vs. MRI surveillance in patients with SCLC (PRIMALung study) [25]. Furthermore, the importance of PCI has been further questioned in the modern immunotherapy era. Recently, the IMpower133 (atezolizumab) [26] and CASPIAN (durvalumab) [27] trials demonstrated an improvement in OS with anti–programmed death ligand 1 antibody therapy when added to platinum-based chemotherapy compared with chemotherapy alone. In IMpower133, 22 (11%) patients in each group received PCI during the maintenance phase. In CASPIAN, PCI was permitted in the platinum-etoposide group only at the investigator’s discretion. Twenty-one (8%) patients in the control group received PCI. The contemporary benefit of PCI in relation to immunotherapy has not been fully elucidated.

The baseline hazard rate function plots of patients with limited-stage SCLC showed differential time-dependent patterns of mortality risks according to the effect of PCI. During the first year of follow-up, the non-PCI group had a higher risk of mortality, while the patterns became comparable again between the two groups. Temporal changes may indicate that the development of immediate brain metastases might be prevented with PCI treatment, thereby leading to short-term survival benefits. Due to the aggressive characteristics of SCLC, which are associated with rapid systemic dissemination, failure to detect metastases at distant sites and newly developed or reseeding brain metastases could inevitably increase risks at a later stage. With the consideration of time-course risk patterns, improved survival outcomes with PCI were observed in patients with limited-stage SCLC.

The current study had several limitations. Due to its retrospective design, selection bias existed despite performing a multivariate analysis. Since we did not have information about objective responses to the initial therapies, we were unable to conduct stratified analyses according to treatment responses. Oncological endpoints other than OS, such as cancer-specific survival, disease-free survival, intracranial relapse, quality of life, and neurocognitive decline after PCI treatment, were not available in this database. Despite the importance of positron emission tomography (PET)/computed tomography (CT) in the clinical staging of SCLC [28], an accurate number of patients diagnosed with the use of PET/CT scans was not available in the registry. Regarding the multi-institutional study design, heterogeneity in treatment policy was inevitable. In particular, we recognized that a high proportion of patients did not receive CRT, and undertreatment in patients who did not receive CRT and/or PCI was a potential source of bias. Although multidisciplinary discussion is essential for the treatment of SCLC, the primary treatment decision is mainly made by medical oncologists in Korea, and sometimes the treatment strategy is dependent on a physician’s discretion. A retrospective study from a leading hospital in Korea, Asan Medical Center, observed a heterogeneous pattern in the use of PCI in clinical practice, which is similar to our observation. The authors reported that approximately 54% of limited-stage SCLC patients with partial response or complete response after RT were not treated with PCI [29]. In this sense, the results of our study may provide sufficient grounds for implementing PCI in clinical practice. Our analysis using data from a national registry was informative to support the efficacy of PCI in current clinical practice based on contemporary guidelines.

In conclusion, PCI might be beneficial for the survival of patients with limited-stage SCLC who have no brain metastases at diagnosis but not for those with extensive-stage disease. According to the time-course hazard rate function plots, patients with limited-stage SCLC who did not receive PCI had a higher mortality risk during the first year of follow-up than those who received PCI, thereby indicating the prognostic implications of PCI in the prevention of immediate intracranial failure after definitive treatment. In accordance with the aforementioned Japanese trial, routine adoption of PCI treatment is not required for patients with extensive-stage SCLC in the modern era of brain MRI staging. The role of PCI needs to be reassessed regarding current trends in diagnostic and treatment practice.

## Supplementary Information

**Additional file 1.** **Supplementary Materials:** Details of treatment. **Table S1:** Clinical and treatment-related characteristics of patients with extensive-stage small cell lung cancer without brain metastases at baselin. **Table S2:** Prognostic factors in patients with extensive-stage disease without brain metastases at baseline.

## Data Availability

The datasets of the current study are available from the corresponding author upon reasonable request.

## Abbreviations

MRI: Magnetic resonance imaging
PCI: Prophylactic cranial irradiation
SCLC: Small-cell lung cancer
OS: Overall survival
AJCC: American Joint Committee on Cancer
EORTC: European Organisation for Research and Treatment of Cancer
CT: Computed tomography
PET: Positron emission tomography
ECOG: Eastern Cooperative Oncology Group
BMI: Body mass index
RT: Radiotherapy
CRT: Chemoradiotherapy
CI: Confidence interval
HR: Hazard ratio
PFS: Progression-free survival
