# Supplementary material for: Survival impact of prophylactic cranial irradiation in small-cell lung cancer in the modern era of magnetic resonance imaging staging
Source: Radiat Oncol. 2022 Feb 5;17:26. doi: 10.1186/s13014-022-01994-8 (PMC8817587; doi:10.1186/s13014-022-01994-8)
Supplement: Supplementary file 1 — Additional file 1. Supplementary Materials: Details of treatment. Table S1: Clinical and treatment-related characteristics of patients with extensive-stage small cell lung cancer without brain metastases at baselin. Table S2: Prognostic factors in patients with extensive-stage disease without brain metastases at baseline. [file 13014_2022_1994_MOESM1_ESM.docx]

**Appendix 1: Supplementary Materials**

**Details of treatment**

First-line chemotherapy mainly consisted of cisplatin and carboplatin-based doublets, represented by cisplatin-etoposide and carboplatin-etoposide regimens. The median cycles of initial chemotherapy were 4 (range, 1‒18) and 6 (range, 1‒18) for limited- and extensive-stage, respectively. Regarding thoracic radiotherapy (RT), the median daily and total radiation dose were 2 Gy (range, 1.5‒15 Gy) and 54 Gy (range, 30‒68 Gy), respectively, for the limited-stage group, and 2 Gy (range, 1.5‒5.0 Gy) and 50.4 Gy (range, 25‒66 Gy), respectively, for the extensive-stage group. The three-dimensional conformal RT technique was used in approximately 67% of the thoracic RT cases in the limited-stage group and 50% in the extensive-stage group.

Salvage treatment included chemoradiotherapy, chemotherapy, RT, and surgery. Approximately 38% (n = 41) of limited- and 43% (n = 52) of extensive-stage patients received the subsequent lines of treatment. Of this population, chemoradiotherapy and chemotherapy accounted for the majority of treatment, as 31 patients in the limited-stage group and 40 in the extensive-stage group received these therapies.

**Appendix 2: Supplementary Tables**

**Table S1.** Clinical and treatment-related characteristics of patients with extensive-stage who had no brain metastases at baseline

| Variables | Number of patients (%) | | *P* |
| --- | --- | --- | --- |
|  | PCI  (n = 17) | No PCI  (n = 105) |  |
| Age (year) |  |  |  |
| Median (range) | 64 (51‒74) | 69 (32‒91) | 0.061 |
| Gender |  |  |  |
| Male | 15 (88) | 89 (85) | 1.000 |
| Female | 2 (12) | 16 (15) |  |
| ECOG performance score^a^ |  |  |  |
| 0‒1 | 16 (94) | 66 (63) | 0.182 |
| ≥ 2 | 1 (6) | 19 (18) |  |
| Ever-smoker^a^ |  |  |  |
| No | 1 (6) | 26 (25) | 0.117 |
| Yes | 15 (94) | 78 (74) |  |
| Body mass index (kg/m^2^) |  |  |  |
| Median (range) | 23.1 (20.5‒31.9) | 22.8 (17.1‒32.8) | 0.744 |
| No. of symptoms at diagnosis |  |  |  |
| Median (range) | 2 (0‒4) | 1 (0‒5) | 0.117 |
| Clinical T stage^a^ |  |  |  |
| T1‒2 | 3 (18) | 24 (23) | 0.751 |
| T3‒4 | 11 (65) | 58 (55) |  |
| Clinical N stage^a^ |  |  |  |
| N0‒1 | 0 (0) | 5 (5) | 1.000 |
| N2‒3 | 17 (100) | 92 (95) |  |
| Extrathoracic metastasis |  |  |  |
| No | 2 (12) | 30 (29) | 0.144 |
| Yes | 15 (88) | 75 (71) |  |
| Definitive treatment |  |  |  |
| Chemoradiotherapy^b^ | 8 (47) | 7 (7) | < 0.001 |
| Chemotherapy | 8 (47) | 91 (86) |  |
| Others^c^ | 1 (6) | 7 (7) |  |
| Chemotherapy regimens |  |  |  |
| Cisplatin doublet | 12 (75) | 54 (60) | 0.262 |
| Carboplatin doublet | 3 (19) | 34 (38) |  |
| Others | 1 (6) | 2 (2) |  |
| Cycles of first-line chemotherapy |  |  |  |
| < 4 | 2 (12) | 36 (40) | 0.047 |
| ≥ 4 | 14 (87) | 54 (60) |  |
| Salvage treatment |  |  |  |
| Chemoradiotherapy^b^ | 4 (24) | 14 (13) | 0.070 |
| Chemotherapy | 6 (35) | 16 (15) |  |
| Others^c^ | 2 (12) | 10 (10) |  |
| No treatment | 5 (29) | 65 (62) |  |

^a^Missing values were excluded.

^b^Cases with concurrent or sequential chemoradiotherapy were included.

^c^Patients who received radiotherapy alone or surgery (+/- adjuvant therapy) were included.

PCI, prophylactic cranial irradiation; ECOG, Eastern Cooperative Oncology Group.

**Table S2.** Multivariate analysis for overall survival in patients with extensive-stage who had no brain metastases at baseline

| Variables | Multivariate analysis | | |
| --- | --- | --- | --- |
|  | Hazard ratio | 95% CI | *P* |
| Age (years)^a^ |  |  |  |
| ≤ 68 | Ref |  |  |
| > 68 | 1.69 | 1.08‒2.64 | 0.023 |
| ECOG score |  |  |  |
| 0‒1 | Ref |  |  |
| ≥ 2 | 2.11 | 1.21‒3.66 | 0.008 |
| Body mass index (kg/m^2^)^a^ |  |  |  |
| ≥ 23.6 | Ref |  |  |
| < 23.6 | 1.37 | 0.87‒2.17 | 0.179 |
| Clinical N stage |  |  |  |
| N0‒1 | Ref |  |  |
| N2‒3 | 2.21 | 0.64‒7.64 | 0.209 |
| Salvage treatment |  |  |  |
| Chemoradiotherapy^b^ | Ref |  |  |
| Others^c^ | 1.30 | 0.54‒3.12 | 0.556 |
| Chemotherapy | 2.66 | 1.27‒5.57 | 0.010 |
| No treatment | 4.09 | 2.19‒7.62 | < 0.001 |

^a^The median value was used as the cutoff point.

^b^Cases with concurrent or sequential chemoradiotherapy were included.

^c^Patients who received radiotherapy alone or surgery (+/- adjuvant therapy) were included.

CI, confidence interval; Ref, reference.
